# Functional Invariant Natural Killer T Cells Secreting Cytokines Are Associated With Non-Progressive Human Immunodeficiency Virus-1 Infection but Not With Suppressive Anti-Retroviral Treatment

**DOI:** 10.3389/fimmu.2018.01152

**Published:** 2018-05-24

**Authors:** Dharmendra Singh, Manisha Ghate, Sheela Godbole, Smita Kulkarni, Madhuri Thakar

**Affiliations:** ^1^Department of Immunology, National AIDS Research Institute, Pune, India; ^2^Department of Clinical Sciences, National AIDS Research Institute, Pune, India; ^3^Department of Epidemiology and Biostatistics, National AIDS Research Institute, Pune, India; ^4^Department of Virology, National AIDS Research Institute, Pune, India

**Keywords:** invariant natural killer T cells, cytokines, CD1d, long-term non progressors, human immunodeficiency virus

## Abstract

**Background:**

CD1d restricted invariant natural killer T (iNKT) cells are important in the activation and regulation of immune responses. Limited information is available regarding the functional role of iNKT cells in the human immunodeficiency virus (HIV) disease progression.

**Methodology:**

α-GalCer stimulated iNKT cells were characterized for their functionality in terms of cytokine production (IFN-γ, TNF-α, IL-2, IL-4, and IL-21) and CD107a expression in HIV-1 infected [23 long-term non progressors (LTNPs), 28 progressors, 18 patients before and after suppressive anti-retroviral treatment (ART)] along with 25 HIV-1 negative subjects using multicolor flow cytometry.

**Results:**

The functional profile of α-GalCer stimulated iNKT cells was similar in LTNPs and healthy controls. The number of LTNPs showing functional response in terms of secretion of cytokines (IFN-γ/IL2/TNF-α) and CD107a expression was significantly higher than seen in the progressors. The cytokine secretion by the stimulated iNKT cells was predominantly Th1 type. The frequencies of iNKT cells showing secretion of IFN-γ or IL2 or TNF-α or expression of CD107a were higher in LTNPs (*p* < 0.05 for all) and also significantly associated with lower plasma viral load (*p* value ranged from 0.04 to 0.003) and higher CD4 count (*p* value ranged from 0.02 to <0.0001). The functional profile of the iNKT cells before and after ART did not differ significantly indicating absence of restoration of iNKT cells functionality after suppressive ART. The IL-4 and IL-21 secreting iNKT cells were rare in all study populations.

**Conclusion:**

The presence of functional iNKT cells secreting number of cytokines in non-progressive HIV infection could be one of the multiple factors required to achieve HIV control and hence have relevance in understanding the immunity in HIV infection. The failure of restoration of the iNKT functionality after ART should be potential area of future research.

## Introduction

Understanding the innate and acquired immune mechanism in human immunodeficiency virus (HIV) infection is important in designing strategies for prevention of the HIV infection and also for immune therapies in infected individuals. The invariant natural killer T (iNKT) cells are one of the important innate effector cells which get readily activated either upon stimulation of their TCR by CD1d presented glycolipid antigen, or by cytokines in a TCR-independent manner (1, 2) and rapidly produce an array of regulatory and pro-inflammatory cytokines (3, 4), that can subsequently activate and regulate a variety of innate and adaptive immune cells, such as dendritic cells, natural killer cells, and CD4+ and CD8+ T cells (5, 6). These cells have shown to play a role in cancer (7–9), autoimmune diseases (10, 11), and various infectious condition (12–14).

The role of iNKT cells in viral infection has been emphasized by number of studies in mice and humans. The mice deficient in iNKT cells show increased susceptibility or have impaired immune response to several viruses (15). The herpes viruses shown to manipulate CD1d expression to escape iNKT cell surveillance to establish lifelong latency in humans (16). The chronic HIV infection has shown selective depletion of iNKT cells (17–20). The depletion of iNKT cells is reported to be because of either direct infection by HIV as they expressed both CD4 and CCR5 or due to Fas-mediated activation induced cell death (13).

The chronic immune activation is a hallmark of HIV infection. The iNKT cells are known to influence immune activation. Ibarrondo et al. showed that the loss of CD4+ iNKT cells in gut mucosa of HIV-infected individuals was associated with systemic immune activation (21). The iNKT cells known to work early in the disease course as a bridge between innate and acquired immune responses. In HIV infection these cells showed to recognize the HIV-infected DCs early in HIV infection which are then actively targeted by Nef- and Vpu-dependent viral immune evasion mechanism (22). Various studies have reported variable degree of restoration of functionality of these cells after successful anti-retroviral treatment (ART) (23–28). The level of iNKT cell activation in HIV-infected individuals is associated with disease progression and the frequencies and functionality of iNKT cells are preserved in non-progressive HIV infection, such as HIV-1 infected elite controllers and long-term non progressors (LTNPs) (26, 29, 30). After the exposure to SIV, the AIDS resistant mangabeys also showed higher frequency of iNKT cells as compared to the SIV susceptible macaques (31). Our previous study has shown preserved frequencies of iNKT cells with proliferating capacity and lower expression of exhaustive markers in HIV-1-infected LTNPs (26). Since the iNKT cells carry out multiple functions through secretion of number of cytokines, it is important to understand whether this preserved iNKT cell population is also functionally sound. In this study, we assessed the functional characteristics of the iNKT cells in terms of multiple cytokine secretion after stimulation with α-GalCer in individuals with non-progressive HIV infection (LTNPs) and compared with the iNKT cell functionality in progressive HIV infection and also in the individuals with successful ART.

## Materials and Methods

### Study Subjects

23 (9M/14F) LTNPs and 28 (12M/16F) progressors were enrolled from the out-patient clinics of the National AIDS Research Institute, Pune. 18 (10M/8F) of the 28 progressors were initiated on ART. These 18 patients were followed up for 12 months post-ART. The ART was initiated when CD4 count was dropped below 350 cells/mm^3^; as per the National criteria of the ART initiation at the time of enrollment. Additionally 25 (13M/12F) HIV-1 seronegative healthy individuals (HCs) were enrolled in the study. The definitions and clinical details of the study population have been reported earlier (26) and the demographic details of the study participants are given in Table 1.

**Table 1:** Demographic characteristics of study participants.

	Study participants				
HIV status median (IQR)	HCs (*n* = 25)	LTNPs (*n* = 23)	Progressors (*n* = 28)	Anti-retroviral treatment (ART) Naive (*n* = 18)	On ART (12 months) (*n* = 18)
Age (years)	30 (25–38)	36 (33–40)	35 (32–38)	38 (33–41)	39 (34–42)
Viral load (log10)	ND	3.4 (2.4–4.0)	4.5 (3.0–6.2)	4.8 (4.4–5.2)	≤1.60
CD4+ T cells (cells/mm^3^)	937 (696–1,204)	684 (555–1,050)	241 (137–324)	192 (129–293)	372 (286–417)
% CD4 T cells	60 (40–80)	54 (32–78)	36 (10–56)	18 (8–35)	28 (18–39)
% iNKT cells	0.25 (0.12–0.44)	0.16 (0.11–0.25)	0.07 (0.05–0.12)	0.04 (0.03–0.05)	0.08 (0.04–0.12)

*HIV, human immunodeficiency virus; iNKT, invariant natural killer T; ND, not done; IQR, inter quartiles range; HCs, healthy controls; LTNP, long-term non progressors*.

20 ml whole blood sample was collected from each study participants. The plasma and peripheral blood mononuclear cells (PBMCs) were separated as previously described (32), and stored at −80°C and −196°C, respectively until tested. The study was carried out in accordance with the institutional ethics committee (NARI Ethics Committee: Registration No: ECR/23/INST/MH/2013/RR-16). The study (Protocol Number: NARI/EC Protocol No.: 2013-07) was approved by the NARI Ethics committee. All subjects gave written informed consent with the Declaration of Helsinki.

### CD4 Count and Viral Load Estimation

CD4 T cell counts were quantified by Flow cytometry (FACS Calibur, Becton-Dickinson, CA, USA) using TruCount kit (Becton-Dickinson, CA, USA) as described previously (26), and plasma viral load (pVL) was measured as RNA copies/ml by Abbott m2000rt HIV-1 Real-time PCR according to the manufacturer’s instructions.

### Functionality of iNKT Cells

**Figure 1:**
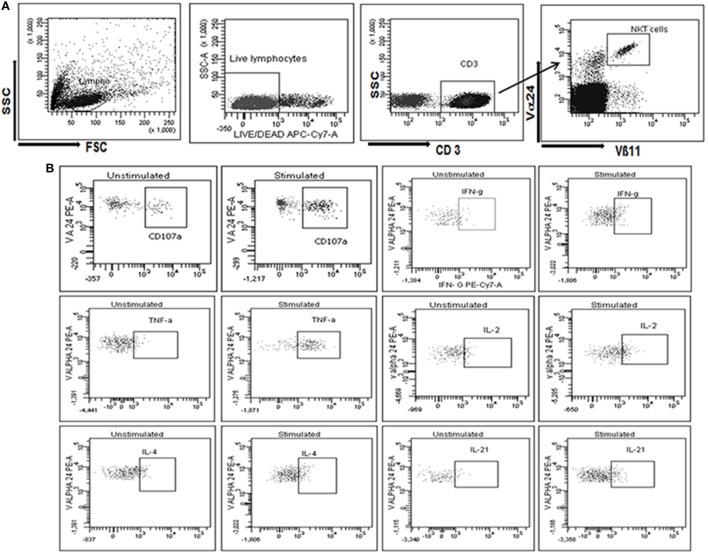
Gating strategy used to assess the identification and functional characterization of invariant natural killer T (iNKT) cells. **(A)** Initially, cells were gated on SSC and FSC followed by gate on live CD3+ T cells population (stained with violet amine reactive dye to identify live cells). The live CD3+ T cells further gated to identify iNKT cells as CD3+, Vα24+, and Vβ11+. **(B)** iNKT cells were further gated to assess the secretion of cytokines, such as IFN-γ, TNF-α, IL-2, IL-4, and/or IL-21 and CD107a expression at unstimulated and stimulated iNKT cells with α-GalCer (**B**1–12 respectively).

The functional capacity of iNKT cells was assessed for intracellular secretion of multiple cytokines, such as IFN-γ, TNF-α, IL-2, and/or IL-4, and CD107a expression after stimulation with α-GalCer using multicolor flow cytometry. Briefly, the cryopreserved PBMCs were revived and rested overnight in RPMI with 10% fetal bovine serum (FBS), at 37°C with 5% CO_2_. The next day, the cells were stimulated with 100 ng/ml α-GalCer (Funakoshi Tokyo, Japan) in the presence of anti-CD107a PerCpCy5.5 (BD Biosciences) for 1 h at 37°C in 5% CO_2_, followed by incubation with Brefeldin A (10 µg/ml; Sigma Aldrich, USA) and GolgiStop (monensin) (1.5 µg/ml; BD Biosciences) for 5 h. Unstimulated cells were also included to assess the background response. The cells were resuspended in 1 ml PBS, washed, and stained with violet amine reactive dye (Invitrogen, Carlsbad, CA, USA) for 30 min at room temperature for differentiation of dead and live cells. The cells were washed and incubated with a cocktail of antibodies; anti-Vα24 PE and anti-Vβ11 FITC (Beckman Coulter, Marseilles, France), anti CD3 PETR (Invitrogen, Carlsbad, CA, USA) for 30 min at room temperature in dark. After washing, the cells were fixed and permeabilized with Perm2 (BD Biosciences, San Jose, CA, USA) according to the manufacturer’s instruction and incubated with a mixture of antibodies; anti-IFN-γ PECy7, anti-TNF-α PECy7, anti-IL-2 APC, anti-IL-4 PerCpCy5.5, and anti-IL-21 APC (all from BD Biosciences, San Jose, CA, USA) for 30 min in the dark at room temperature. The cells were washed and stored at 4°C in the dark until acquisition on FACSAria-I (BD Biosciences, USA) and analyzed using FACSDiva software version 6.1.3 (BD Biosciences, USA). The gating strategy is depicted in Figure 1.

Since the frequency of iNKT cell was very low in PBMCs, particularly in HIV-1-infected individuals, the study participants having more than 0.04% of iNKT cells were included in the functional analysis to ensure an accurate assessment of iNKT cell cytokine production and also for measurement of proliferating ability, expression of exhaustion and senescence markers as previously described (26).

To determine cytokine secreting iNKT cells, lymphocytes were first gated on the basis of forward and side-scatter and second gate was set on live lymphocytes using side scatter and violet amine reactive viability dye. Minimum 100,000 events of live CD3+ T lymphocytes were analyzed. A third gate was set on Vα24+Vβ11+ live lymphocyte and percentage of iNKT cells was calculated from CD3+ live lymphocytes. These iNKT cells were further drilled down to gate on cytokines secreting iNKT cells. Single stained controls were used to set compensation parameters and the unstimulated cells were used to set the gate for cytokine secreting cells.

The background response in unstimulated iNKT cells from the study participants was subtracted from the response shown by stimulated cells and the response above the background level was considered as a response to α-GalCer stimulation.

### Expression of CD57 or PD1+ve iNKT Cells

The expression of immune exhaustion (PD-1) and senescence (CD57) markers was assessed as described previously (26). Briefly, after revival and resting overnight, PBMCs were resuspended in 1 ml PBS and then stained with violet amine reactive dye (Invitrogen, Carlsbad, CA, USA) for 30 min at room temperature for differentiation of dead and live cells. The samples having more than 90% viability were considered for further analysis. The cells were washed again and incubated with a cocktail of antibodies [anti-Vα24 PE, anti-Vβ11 FITC (Beckman Coulter, Marseilles, France), anti-CD57 APC (Biolegend, USA), anti-CD3 PETR (Invitrogen, Carlsbad, CA, USA), anti-CD3 APC, and anti-PD1 PerCpCy5.5 (BD Biosciences)] for 30 min at room temperature. After washing, the cells were fixed in 3% paraformaldehyde, acquired on FACSAria-I (BD Biosciences, USA) and analyzed using FACSAria-I (BD Biosciences, USA) and analyzed using FACSDiva software version 6.1.3 (BD Biosciences, USA).

### Assessment of Proliferation Ability of iNKT Cells

The proliferation ability of α-GalCer stimulated iNKT cells was assessed as described previously (26, 28). Briefly, after revival and resting overnight, 1 × 10^6^ PBMCs were incubated in triplicate in RPMI 1640 with 10% FBS, 100 ng/ml α-GalCer (Funakoshi Tokyo, Japan), and 50 IU/ml recombinant human IL-2 (Roche Diagnostics, USA). The medium was replenished at day 3 and 7, and the culture was analyzed for iNKT cells frequency at day 0 and 13 on flow cytometry as described previously (26, 28).

### Statistical Analysis

GraphPad Prism version 5.01 software was used for statistical analyses. Differences in variables between the study groups were analyzed with Mann–Whitney *U* test and spearman test was used for the correlation analysis. The mean of triplicate experiments for proliferation assessment was considered for the analysis. Changes in the parameter over the time (before and after ART) were analyzed with paired *t*-test. *p* Value of <0.05 was considered as significant.

## Results

### Cytokine Secretion Profile of iNKT Cells in LTNPs Was Similar to That Seen in Healthy Controls

The ability of α-GalCer stimulated iNKT cells from HIV uninfected and infected individuals to secrete IFN-γ, IL-2, TNF-α, IL-4, or IL-21 was assessed along with their ability to express CD107a as a marker of cytotoxicity using flow cytometry. We observed that the iNKT cells from 24 out of 25 (96%) HCs, 22 of 23 LTNPs (95.65%) and 20 of 28 progressors (71.46%), and 11 of 18 ART-treated (61.11%) individuals responded to α-GalCer stimulation and secreted one or more cytokines.

**Figure 2:**
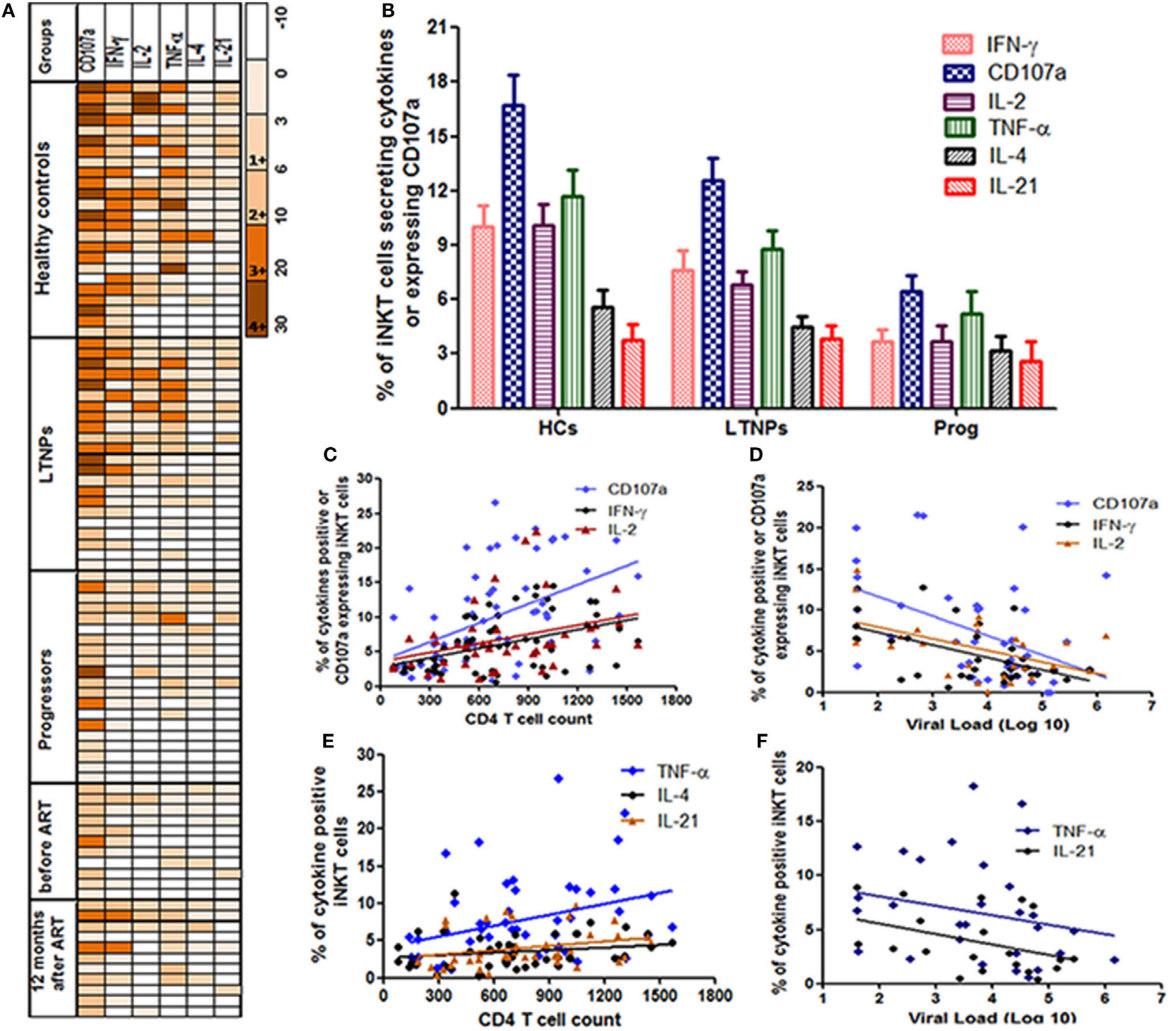
Assessment of the cytokines secretion profile of invariant natural killer T (iNKT) cells. **(A)** Heatmaps of iNKT cells secreting cytokines (IFN-γ, IL-2, TNF-α, IL-4, or IL-21) or expressing CD107a from all study groups [HCs, long-term non progressors (LTNPs), progressors, before, and post anti-retroviral treatment at 12 months]. Only responders are represented in the heatmaps (each row is single participants). For each particular cytokine, the functional response has been graded from 1+ to 4+ according to the magnitude (% of iNKT cells secreting cytokines or expressing CD107a). **(B)** The bar diagram shows the response (% of iNKT cells showing intracellular cytokine secretion/CD107a expression after stimulation with α-GalCer) on the *Y*-axis in different groups of study participants (HCs, LTNPs, and progressors). **(C)** The scatter plot shows the correlation analysis for percent of IFN-γ+, IL-2+, and CD107a+ iNKT cells (*Y*-axis) with corresponding CD4 T cell counts on the *X*-axis and **(D)** plasma viral load (pVL) (RNA copies/ml) on the *X*-axis. **(E)** The scatter plot shows the correlation analysis for percent of TNF-α+, IL-4+, and IL-21+ iNKT cells (*Y*-axis) with corresponding CD4 T cell counts on the *X*-axis and **(F)** pVL (RNA copies/ml) on the *X*-axis.

Heatmaps of iNKT cells secreting cytokines (IFN-γ, IL-2, TNF-α, IL-4, or IL-21) or expressing CD107a among the responders from all study groups (Figure 2A: each raw is a single participant) demonstrated that LTNPs and HCs showed similar pattern of α-GalCer stimulated cytokine secretion and CD107a expression. The functional profile in HCs (39/144 observations) and LTNPs (24/132 observations) showed higher magnitude of 3+ and 4+ grade (corresponding to 10–20% and 20–30% of iNKT cells secreting particular cytokine or expressing CD107a, respectively) whereas such a high magnitude was rarely observed in the progressor group (5/120 observations). The IL-4 and IL-21 secreting iNKT cells were rare in all study populations (Figure 2A).

The CD107a expression and IFN-γ secretion was found to the most frequent and strong function of iNKT cells from LTNPs and HCs as compared to the progressors. Among the positive responses, the IFN-γ is found to be secreted by the iNKT cells of all HCs (100%), 18/22 LTNPs (81.8%), and 11/20 (55%) progressors. Similarly, among the positive responses the CD107a expressing iNKT cells were observed in 23/24 (95%) HCs, 20/22 LTNPs (90.9%), and 18/20 (90%) progressors. When the magnitude of the functionality (% of stimulated iNKT cells secreting cytokine/s or expressing CD107a) was assessed, the frequencies of IFN-γ secreting or CD107a-expressing iNKT cells were significantly higher in LTNPs as compared to the progressors (*p* < 0.05) but lower than seen in HCs (*p* > 0.05) (Figure 2B).

The frequencies of both IFN-γ secretion and CD107a expression were associated with lower pVL and higher CD4 count in HIV-infected individuals (Figures 2C,F). Overall, the ability to secrete the cytokines or express CD107a was associated with higher CD4 count [IFN-γ+ iNKT cells (*r* = 0.41; *p* = 0.0016), CD107a+ iNKT cells (*r* = 0.55; *p* < 0.0001), TNF-α+ iNKT cells (*r* = 0.37; *p* = 0.008), IL-4+ iNKT cells (*r* = 0.30; *p* = 0.04), IL-2+ iNKT cells (*r* = 0.40; *p* = 0.007), and IL-21+ iNKT cells (*r* = 0.38; *p* = 0.017)] (Figures 2C,D), and lower pVL [IFN-γ+ iNKT cells (*r* = −0.37; *p* = 0.03), for CD107a+ iNKT cells (*r* = −0.37; *p* = 0.01), IL2+ iNKT cells (*r* = −0.39; *p* = 0.04), and for TNF-α+ iNKT cells (*r* = −0.38; *p* = 0.03)] (Figures 2E,F), whereas no significant association was observed with IL-4+ iNKT cells (*r* = −0.19; *p* = 0.38) or IL-21+ iNKT cells (*r* = −0.24; *p* = 0.1).

### The Functionality of iNKT Cells Was Not Restored After Suppressive ART

The pattern of α-GalCer stimulated cytokine secretion and CD107a expression was similar in the 18 study participants before and at 12 months after ART (Figure 2A). The iNKT cells secreting IFN-γ/IL-2/TNF-α and/or expressing CD107a were rarely observed (only in 5/18 participants) and the magnitude was always of 1+ or 2+ grade which was not changed significantly after suppressive ART. The grade 3+ and 4+ responses were observed only in case of CD107a expression in three participants and the magnitude of response was changed in case of IFN-γ from grade 2+ to 3+ in two participants (Figure 2A).

**Figure 3:**
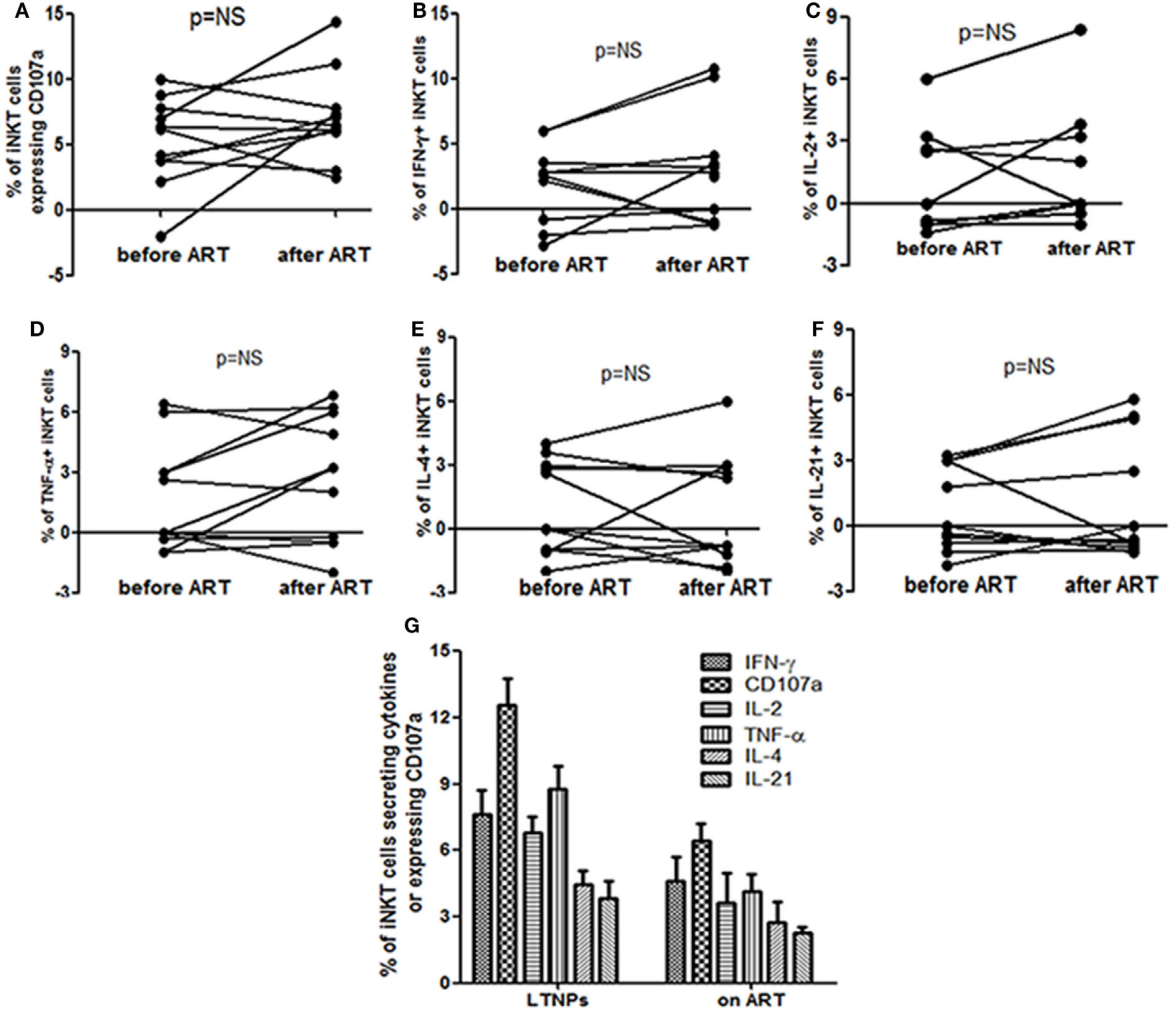
Assessment the impact of anti-retroviral treatment (ART) on the functionality of invariant natural killer T (iNKT) cells: the figure shows percent of **(A)** CD107a+ iNKT cells **(B)**, IFN-γ secreting iNKT cells **(C)**, IL-2 secreting iNKT cells **(D)**, TNF-α secreting iNKT cells **(E)**, IL-4 secreting iNKT cells, and **(F)** IL-21 secreting iNKT before and at 12 months after ART. All the comparisons were done using the Wilcoxon’s signed-rank test on 12 matched samples. **(G)** Comparison of α-GalCer stimulated iNKT cell frequencies secreting IFN-γ, TNF-α, IL-2, IL-4, and/or IL-21 and expressing CD107a on the *Y*-axis in long-term non progressors and at 12 months post ART.

There was no significant increase in cytokine secreting and/or CD107a expressing iNKT cells at 12 months post ART as compared to the percentages before initiation (by Wilcoxon’s signed-rank test) (*p* > 0.05 for all) (Figure 3). The mean frequency of cytokine secreting or CD107a expressing iNKT cells at 12 months post ART was still significantly lower than those observed in LTNPs (*p* > 0.05 to all) (Figure 3). Only IFN-γ+ iNKT cells were found to be significantly associated with higher CD4 counts (*r* = 0.71; *p* = 0.02) at 12 months post ART (data not shown).

### The Cytokine Secretion by iNKT Cells Was Associated With Higher Proliferating Ability of the iNKT Cells

**Figure 4:**
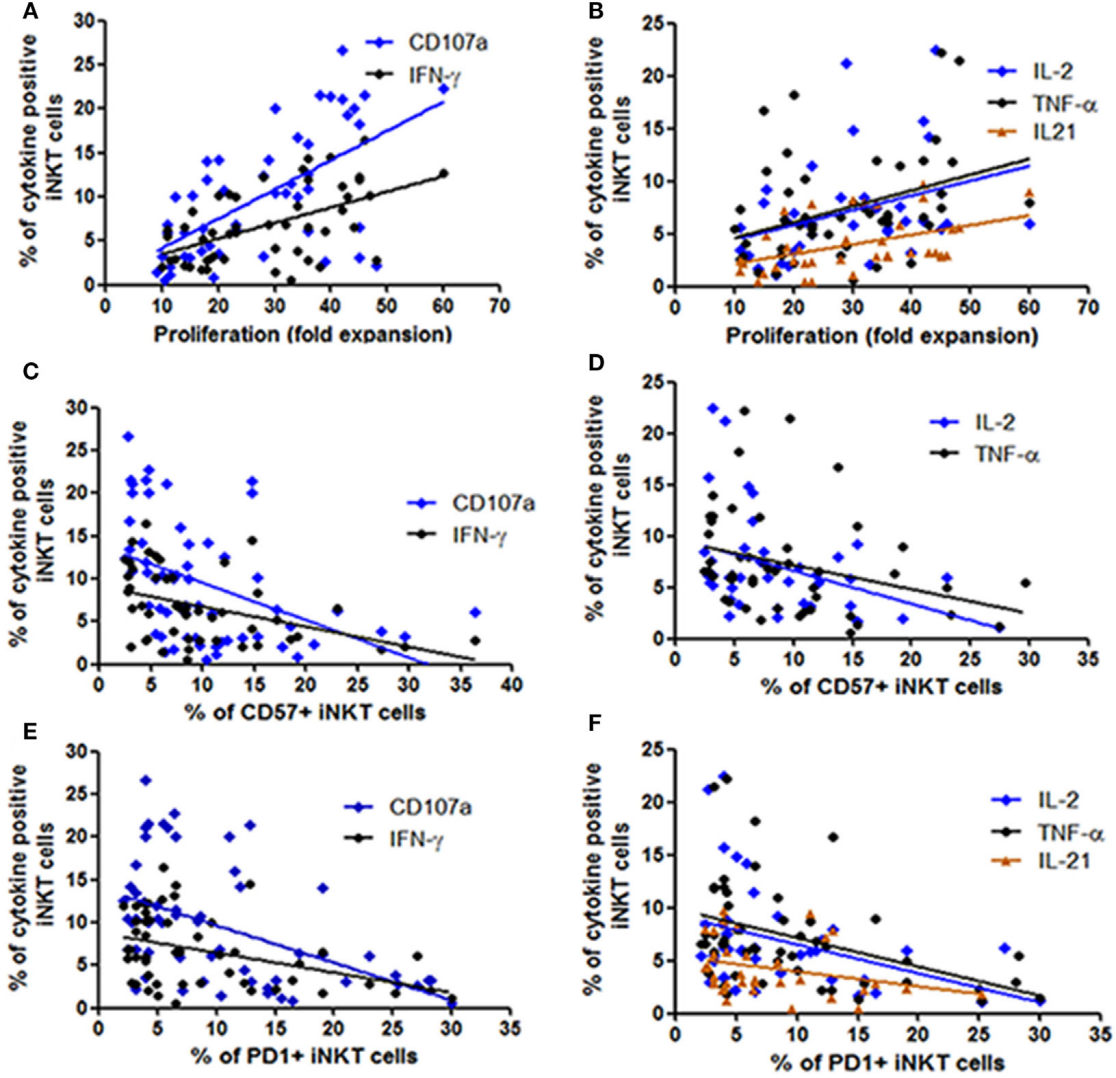
Assessment the relationship between functionality of invariant natural killer T (iNKT) cells with their proliferation ability and expression of immune exhaustion and senescence markers. The scatter diagrams represent the correlation between the percent of iNKT cells secreting cytokines and expressing CD107a on the *Y*-axis with **(A,B)** proliferation ability (fold expansion) of the corresponding iNKT cells on the *X*-axis, **(C,D)** corresponding CD57 expressing iNKT cells on the *X*-axis, and **(E,F)** corresponding PD1 expressing iNKT cells on the *X*-axis.

Earlier we have shown that the iNKT cells from LTNPs have higher proliferating capacity as compared to the iNKT cells from progressors (26). Further, we wanted to assess whether this proliferating ability is associated with the functionality of iNKT cells. As expected, the proliferating ability was significantly associated with the higher frequencies of cytokine secreting and degranulating iNKT cells [IFN-γ+ iNKT cells (*r* = 0.37; *p* < 0.0001), CD107a+ iNKT cells (*r* = 0.58; *p* < 0.0001), TNF-α+ iNKT cells (*r* = 0.37; *p* = 0.028), IL-2+ iNKT cells (*r* = 0.47; *p* = 0.01), and IL-21 + iNKT cells (*r* = 0.55; *p* = 0.003)] (Figures 4A,B).

### CD57+ and PD1 Expression Was Associated With Poor Functionality

In our previous study in the same study population we observed that the progressive HIV infection is associated with higher expression of CD57 and PD1 expressing iNKT cells (26). We observed that the frequencies of both CD57 or PD1 expressing iNKT cells were associated with lower frequencies of cytokine secreting and degranulating iNKT cells [in case of CD57 expressing iNKT cells: for IFN-γ+ iNKT (*r* = −0.47; *p* = 0.001), for CD107a+ iNKT cells (*r* = −0.55; *p* < 0.0001), for IL-2+ iNKT cells (*r* = −0.38; *p* = 0.01), for TNF-α+ iNKT cells (*r* = −0.41; *p* = 0.002) (Figures 4C,D), and in case of PD-1-expressing iNKT cells for IFN-γ+ iNKT (*r* = −0.49; *p* = 0.004), for CD107a+ iNKT cells (*r* = −0.50; *p* < 0.0001), for IL-2+ iNKT cells: *r* = −0.33; *p* = 0.02, for TNF-α+ iNKT cells (*r* = −0.44; *p* = 0.001), and for IL-21 + iNKT cells (*r* = −0.35; *p* = 0.03)] (Figures 4E,F).

## Discussion

The present study indicate that the multiple cytokine secreting functional iNKT cells are associated with non-progressive HIV infection and the pattern of cytokine secretion is similar with that seen in the HIV negative healthy controls. We used LTNPs as a model of non-progressive HIV infection to assess the functionality of α-GalCer stimulated iNKT cells in HIV infection. Since iNKT cells can regulate both adaptive and innate immune responses through the rapid production of a vast array of cytokines, we determined the functionality of iNKT cells in terms of secretion of multiple cytokines and expression of CD107a as a marker of cytotoxicity. The heatmap analysis showed similar pattern of cytokine secretion by the iNKT cells from LTNPs and healthy controls after α-GalCer stimulation. This finding supports our previous observation of preserved frequencies of iNKT cells in non-progressive HIV infection (26) and further confirms that the iNKT cells retain the functionality in terms of the multiple cytokine secretion and cytotoxicity in LTNPs. Our observation of higher number of functional iNKT cells secreting multiple cytokines in non-progressive HIV infection contributes to the available information on HIV pathogenesis stating that the functional innate effector response could also be important in virus control in HIV infection. Further, the cytokine response was predominantly Th1 type as the IFN-γ and IL-2 secretion were the most frequently observed functions of α-GalCer stimulated iNKT cells with a weak IL-4 secretion. The mucosal iNKT cells secreting IL-4 (Th2 type) were shown be associated with lower immune activation (29). The differences in the observation might be due to the analysis of iNKT cells from different sites. The Th1 iNKT cells have been shown to have better prognosis in chronic lymphocytic leukemia (33). The observation in this study highlighted the importance of Th1 profile of iNKT cells in chronic infection-like HIV. The predominant secretion of Th1 cytokine could induce potent CD8 and NK cell response resulting in killing of HIV-infected cells. We observed proliferating ability of iNKT cells was associated with higher cytokine secretion whereas the CD57 and PD-1 expressing iNKT cell frequencies were associated with reduced ability to secrete cytokines. Hence although it was an expected phenomenon for iNKT cells, the presence of lower expression of CD57 and PD-1 on iNKT cells with more proliferating and cytokine inducing ability indicates the sound and competent immune response in LTNPs which might be responsible for efficient virus control and halting the disease progression. It might also be possible that the functional iNKT cells help in facilitating the antitumor activity in HIV infection. The PD-1 blockade using anti PD-1 antibodies along with α-GalCer has shown improvement in iNKT cell functions leading to persistent anti-metastasis response in mouse model (34). Similar strategy can be worth exploring in case of HIV infection.

The ART has shown to have a profound benefit in improving the HIV control, quality of life and life expectancy. The early ART initiation has shown better restoration of iNKT cells frequencies (23, 27) and functionality (24). However, in our study the ART has been initiated late in the course of infection (CD4 < 350), it could be the reason for no improvement in the functionality of the iNKT cells even after suppressive ART for 12 months (Figures 2A and 3). Previously we have shown that the ART in the same study population partially restored the quantity of iNKT cells (26). It would be interesting to explore the functionality of iNKT cells in the LTNPs who are initiated on ART in the test and treat era. It is possible that the failure of restoration of iNKT functionality after ART might lead to susceptibility of HIV-associated cancers and other infections. Although the benefits of ART could not undermine the strategies to improve the iNKT functionality with ART would be worth exploring.

In conclusion, our observation of presence of functional iNKT cells secreting IFN-γ, IL-2, and having degranulating ability in non-progressive HIV infection in indicator of importance of sound immune system in achieving HIV control. The restoration of the iNKT functionality after ART could be potential area of future research especially considering the long-term benefits of ART.

## Ethics Statement

The study was carried out in accordance with the institutional ethics committee (NARI Ethics Committee: Registration No: ECR/23/INST/MH/2013/RR-16). The study (Protocol Number: NARI/EC Protocol No.: 2013-07) was approved by the NARI Ethics committee. All subjects gave written informed consent with the Declaration of Helsinki.

## Author Contributions

DS designed the protocol and performed all the lab experiments. MG and SG developed the enrollment criteria, sample collection, and follow up the samples. SK has been done viral load testing and data analysis. MT and DS designed the hypothesis and manuscript writing and data analysis. MT has been planned and monitored of all experiments of the study. All authors read and approved the manuscript.

## Conflict of Interest Statement

The authors declare that the research was conducted in the absence of any commercial or financial relationships that could be construed as a potential conflict of interest.

## References

[1] Brigl M Bry L Kent SC Gumperz JE Brenner MB. Mechanism of CD1d-restricted natural killer T cell activation during microbial infection. Nat Immunol (2003) 4(12):1230–7. 10.1038/ni1002 14578883

[2] Mattner J Debord KL Ismail N Goff RD Cantu C Zhou D Exogenous and endogenous glycolipid antigens activate NKT cells during microbial infections. Nature (2005) 434(7032):525–9. 10.1038/nature03408 15791258

[3] Mattarollo SR Yong M Tan L Frazer IH Leggatt GR Secretion of IFN-gamma but not IL-17 by CD1d-restricted NKT cells enhances rejection of skin grafts expressing epithelial cell-derived antigen. J Immunol (2010) 184(10):5663–9. 10.4049/jimmunol.0903730 20410490 PMC2921625

[4] Godfrey DI Stankovic S Baxter AG. Raising the NKT cell family. Nat Immunol (2010) 11(3):197–206. 10.1038/ni.1841 20139988

[5] Carnaud C Lee D Donnars O Park SH Beavis A Koezuka Y Cutting edge: cross-talk between cells of the innate immune system: NKT cells rapidly activate NK cells. J Immunol (1999) 163(9):4647–50. 10528160

[6] Hermans IF Silk JD Gileadi U Salio M Mathew B Ritter G NKT cells enhance CD4+ and CD8+ T cell responses to soluble antigen in vivo through direct interaction with dendritic cells. J Immunol (2003) 171(10):5140–7. 10.4049/jimmunol.171.10.5140 14607913

[7] Nowicki MJ Vigen C Mack WJ Seaberg E Landay A Anastos K Association of cells with natural killer (NK) and NKT immunophenotype with incident cancers in HIV-infected women. AIDS Res Hum Retroviruses (2008) 24(2):163–8. 10.1089/aid.2007.0119 18240964

[8] Berzofsky JA Terabe M. NKT cells in tumor immunity: opposing subsets define a new immunoregulatory axis. J Immunol (2008) 180(6):3627–35. 10.4049/jimmunol.180.6.3627 18322166

[9] Metelitsa LS Naidenko OV Kant A Wu HW Loza MJ Perussia B Human NKT cells mediate antitumor cytotoxicity directly by recognizing target cell CD1d with bound ligand or indirectly by producing IL-2 to activate NK cells. J Immunol (2001) 167(6):3114–22. 10.4049/jimmunol.167.6.3114 11544296

[10] Wu L Van Kaer L Natural killer T cells and autoimmune disease. Curr Mol Med (2009) 9(1):4–14. 10.2174/156652409787314534 19199937

[11] Ronchi F Falcone M. Immune regulation by invariant NKT cells in autoimmunity. Front Biosci (2008) 13:4827–37. 10.2741/3042 18508548

[12] Fernandez CS Kelleher AD Finlayson R Godfrey DI Kent SJ. NKT cell depletion in humans during early HIV infection. Immunol Cell Biol (2014) 92(7):578–90. 10.1038/icb.2014.25 24777308

[13] Vasan S Tsuji M. A double-edged sword: the role of NKT cells in malaria and HIV infection and immunity. Semin Immunol (2010) 22(2):87–96. 10.1016/j.smim.2009.11.001 19962909 PMC3603358

[14] Snyder-Cappione JE Loo CP Carvalho KI Kuylenstierna C Deeks SG Helcht FM Lower cytokine secretion ex vivo by natural killer T cells in HIV-infected individuals is associated with higher CD161 expression. AIDS (2009) 23(15):1965–70. 10.1097/QAD.0b013e32832b5134 19590406

[15] Diana J Lehuen A. NKT cells: friend or foe during viral infections? Eur J Immunol (2009) 39(12):3283–91. 10.1002/eji.200939800 19830742

[16] Chung BK Priatel JJ Tan R. CD1d expression and invariant NKT cell responses in herpesvirus infections. Front Immunol (2015) 6:312. 10.3389/fimmu.2015.00312 26161082 PMC4479820

[17] Motsinger A Haas DW Stanic AK Van Kaer L Joyce S Unutmaz D. CD1d-restricted human natural killer T cells are highly susceptible to human immunodeficiency virus 1 infection. J Exp Med (2002) 195(7):869–79. 10.1084/jem.20011712 11927631 PMC2193731

[18] Sandberg JK Fast NM Palacios EH Fennelly G Dobroszycki J Palumbo P Selective loss of innate CD4(+) V alpha 24 natural killer T cells in human immunodeficiency virus infection. J Virol (2002) 76(15):7528–34. 10.1128/JVI.76.15.7528-7534.2002 12097565 PMC136353

[19] van der Vliet HJ von Blomberg BM Hazenberg MD Nishi N Otto SA Van Benthem BH Selective decrease in circulating V alpha 24+V beta 11+ NKT cells during HIV type 1 infection. J Immunol (2002) 168(3):1490–5. 10.4049/jimmunol.168.3.1490 11801694

[20] Crowe NY Godfrey DI Baxter AG Natural killer T cells are targets for human immunodeficiency virus infection. Immunology (2003) 108(1):1–2. 10.1046/j.1365-2567.2003.01580.x 12519295 PMC1782857

[21] Ibarrondo FJ Wilson SB Hultin LE Shih R Hausner MA Hultin PM Preferential depletion of gut CD4-expressing iNKT cells contributes to systemic immune activation in HIV-1 infection. Mucosal Immunol (2013) 6(3):591–600. 10.1038/mi.2012.101 23149661 PMC3865278

[22] Paquin-Proulx D Gibbs A Bachle SM Checa A Introini A Leeansyah E Innate invariant NKT cell recognition of HIV-1-infected dendritic cells is an early detection mechanism targeted by viral immune evasion. J Immunol (2016) 197(5):1843–51. 10.4049/jimmunol.1600556 27481843 PMC4991248

[23] van der Vliet HJ van Vonderen MG Molling JW Bontkes HJ Reijm M Reiss P Cutting edge: rapid recovery of NKT cells upon institution of highly active antiretroviral therapy for HIV-1 infection. J Immunol (2006) 177(9):5775–8. 10.4049/jimmunol.177.9.5775 17056500

[24] Vasan S Poles MA Horowitz A Siladji EE Markowitz M Tsuji M. Function of NKT cells, potential anti-HIV effector cells, are improved by beginning HAART during acute HIV-1 infection. Int Immunol (2007) 19(8):943–51. 10.1093/intimm/dxm055 17702988

[25] Yang OO Wilson SB Hultin LE Detels R Hultin PM Ibarrondo FJ Delayed reconstitution of CD4+ iNKT cells after effective HIV type 1 therapy. AIDS Res Hum Retroviruses (2007) 23(7):913–22. 10.1089/aid.2006.0253 17678476

[26] Singh D Ghate M Godbole S Kulkarni S Thakar M. CD1d-restricted natural killer T cells are preserved in Indian long-term nonprogressors. J Acquir Immune Defic Syndr (2017) 75(4):e104–12. 10.1097/QAI.0000000000001322 28650939

[27] Moll M Snyder-Cappione J Spotts G Hecht FM Sandberg JK Nixon DF. Expansion of CD1d-restricted NKT cells in patients with primary HIV-1 infection treated with interleukin-2. Blood (2006) 107(8):3081–3. 10.1182/blood-2005-09-3636 16368878 PMC1895745

[28] Moll M Kuylenstierna C Gonzalez VD Andersson SK Bosnjak L Sonnerborg A Severe functional impairment and elevated PD-1 expression in CD1d-restricted NKT cells retained during chronic HIV-1 infection. Eur J Immunol (2009) 39(3):902–11. 10.1002/eji.200838780 19197939 PMC2736548

[29] Paquin-Proulx D Ching C Vujkovic-Cvijin I Fadrosh D Loh L Huang Y Bacteroides are associated with GALT iNKT cell function and reduction of microbial translocation in HIV-1 infection. Mucosal Immunol (2017) 10(1):69–78. 10.1038/mi.2016.34 27049061 PMC5053825

[30] Bachle SM Malone DF Buggert M Karlsson AC Isberg PE Biague AJ Elevated levels of invariant natural killer T-cell and natural killer cell activation correlate with disease progression in HIV-1 and HIV-2 infections. AIDS (2016) 30(11):1713–22. 10.1097/QAD.0000000000001147 27163705 PMC4925311

[31] Rout N Greene J Yue S O’Connor D Johnson RP Else JG Loss of effector and anti-inflammatory natural killer T lymphocyte function in pathogenic simian immunodeficiency virus infection. PLoS Pathog (2012) 8(9):e1002928. 10.1371/journal.ppat.1002928 23028326 PMC3447755

[32] Kulkarni AG Paranjape RS Thakar MR. Higher expression of activating receptors on cytotoxic NK cells is associated with early control on HIV-1C multiplication. Front Immunol (2014) 5:222. 10.3389/fimmu.2014.00222 24904577 PMC4032894

[33] Gorini F Azzimonti L Delfanti G Scarfò L Scielzo C Bertilaccio MT Invariant NKT cells contribute to chronic lymphocytic leukemia surveillance and prognosis. Blood (2017) 129(26):3440–51. 10.1182/blood-2016-11-751065 28465341

[34] Durgan K Ali M Warner P Latchman YE. Targeting NKT cells and PD-L1 pathway results in augmented anti-tumor responses in a melanoma model. Cancer Immunol Immunother (2011) 60(4):547–58. 10.1007/s00262-010-0963-5 21240487 PMC3207499

